# Feasibility of the EQ-5D in the elderly population: a systematic review of the literature

**DOI:** 10.1007/s11136-021-03007-9

**Published:** 2021-10-06

**Authors:** Ole Marten, Laura Brand, Wolfgang Greiner

**Affiliations:** Department of Health Economics and Health Care Management, School of Public Health, Bielefeld University, Universitaetsstrasse 25, Bielefeld, Germany

**Keywords:** EQ-5D, EQ-5D-5L, Health-related quality of life, Systematic review, Feasibility, Elderly

## Abstract

**Purpose:**

The EQ-5D-3L and 5L are widely used generic preference-based instruments, which are psychometrically sound with the general population, but little is known about the instruments’ feasibility in the elderly. Therefore, this systematic review summarises the available literature with regard to the feasibility properties of the instruments in the elderly population.

**Methods:**

We conducted a systematic search in PubMed, PsycInfo and EuroQol databases using pre-specified vocabulary and inclusion/exclusion criteria to identify publications until November 2020. Study characteristics and outcomes referring to the feasibility of the EQ-5D-3L and 5L in the elderly were extracted, if all study participants were at least 65+ years.

**Results:**

We identified 17 studies reporting feasibility outcomes based on four criteria: missing values, completion rates, completion time and broad qualitative statements referring to the completion. Missing values per dimension ranged from 0 to 10.7%, although being mostly below 7%. The completion rate was around 90% or better, whereas the EQ VAS rating was missing from 2.3 to 25.3% of the respondents. Only two of the included studies examined the EQ-5D-5L; 15 studies reported on the EQ-5D-3L.

**Conclusion:**

Comparing our findings against the general population from published literature, we find that feasibility outcomes in older age groups are just below that of younger populations. Furthermore, older respondents have a higher propensity of requiring assistance or even an interviewer-based approach. Nonetheless, the reviewed literature indicates that the EQ-5D-3L still has good feasibility properties and, hence, is highly applicable in older respondents. However, further research is needed to explore feasibility properties of the EQ-5D-5L in this population.

## Background

In the past decades, the demographic trend of an ageing population has become one of society’s central challenges. Especially the proportion of the elderly population, i.e. people being 65 years and above [1], is growing faster than any other age group [2]. At the same time, the increase in life expectancy induces a higher individual risk of contracting one or multiple diseases over the course of life, eventually resulting in a growing number of multi-morbid patients with chronic diseases [3]. Chronic illness and multi-morbidity are known to be associated with disability, declined functional status and diminished quality of life (QoL) [4], which is further linked to higher health care utilisation and increased costs of health care [5]. Given the natural limit of health care resources, it is necessary to evaluate the cost-effectiveness of health care interventions from a societal perspective to sustain the health service provision [6].

Health-related quality of life (HRQoL) is a central outcome for the benefit assessment of health and social care interventions often measured as patient reports to gather the patients’ subjective assessment of their health condition. The EQ-5D is a generic preference-based measure of HRQoL to operationalise quality-adjusted life years (QALYs) in economic evaluation [7]. The EQ-5D consists of two sections. The first is a descriptive system covering five dimensions: mobility (MO), self-care (SC), usual activities (UA), pain or discomfort (PD) and anxiety or depression (AD). In the EQ-5D-3L each dimension can be described by three severity levels (1—no problems; 2—moderate problems; 3—unable to), thus allowing to distinguish 243 unique health states (3^5). The second part is a visual analogue scale (EQ VAS)—a vertical thermometer—ranging from 0 (‘worst imaginable health’) to 100 (‘best imaginable health’) gauging the respondent’s subjectively rated health which might cover aspects different from those in the descriptive system [8, 9]. A later variant, the EQ-5D-5L, covers the same five dimensions, but allowing the respondent to choose from five response levels, thus describing 3125 unique health states [10]. For both versions of the EQ-5D the descriptive system can be scored using a tariff, which provides the preference weights for each health state allowing to calculate an index value on the 0–1 QALY scale, whereas the EQ VAS rating is commonly analysed independently from the responses to the descriptive system [11].

The EQ-5D (hereafter, used to refer to both the EQ-5D-3L and EQ-5D-5L) has been shown to be applicable in various health conditions and populations and is the most widely used instrument for use in economic evaluation [12–14]. Literature suggest that the EQ-5D is also frequently used in the economic evaluation of interventions for the elderly population [15–19]. However, as of yet there is no established gold standard as to how HRQoL in the elderly should ultimately be measured. Especially with regard to the elderly population further requirements to the appropriate assessment of HRQoL are made, since people of higher age may be different to their younger counterparts in the general population in terms of their physical or mental abilities, education or understanding of health [17, 20]. Common criteria to evaluate an instrument’s performance are its reliability, validity as well as its feasibility [21]. Generally, the EQ-5D’s measurement properties are well examined [22, 23] with several studies specifically confirming the instrument’s reliability and validity in the older population, however, leaving the feasibility property widely untouched and warranting further research [18, 24, 25]. In this sense, feasibility is concerned with the difficulty or ease of applying the measure in a population, which translates into how well the measure is regularly completed [21, 26, 27]. Beyond these descriptions there is no gold standard to the definition or operationalisation of feasibility with regard to HRQoL measures. A prior search across all age groups indicates that the feasibility of the EQ-5D is typically associated with the proportion of missing values [13, 28–31], time required for completion and the appropriateness of the administration mode [26, 32].

Age-related decline may be an obstacle for elderly respondents when self-reporting their HRQoL in a survey [33, 34]. Therefore, offering interviewer support or administration can help to reduce the burden to respondents and thereby have a positive impact on item response [35, 36]. There is recent qualitative evidence from older (60+ years) hip fracture patients suggesting that interviewer support was needed and had a positive effect on the instruments’ completion [37]. Even though the EQ-5D is a short measure there appears to be some demand for interviewer support, however, it is unclear how commonly this is applied when collecting EQ-5D data in the elderly.

Despite the steadily growing population of those aged 65 years and above, to date little is known about the feasibility of the EQ-5D in the elderly. Therefore, the aims of this literature review were as follows: (i) to assess the feasibility properties of the EQ-5D in the elderly and (ii) to examine the role of interviewer support in collecting EQ-5D data in samples of the elderly population.

## Methods

### Literature search

We conducted a systematic literature search up until June 2019 with the aim to identify all studies in either English or German assessing the feasibility of the EQ-5D-3L and 5L in the elderly population. We performed electronic searches in the PubMed and PsycINFO (EBSCO) databases as well as the EuroQol Research Foundation Website [38] to identify publications of interest. The search involved MeSH terms and synonymous free-text terms around the following keywords “EQ-5D”, “elderly” and “feasibility”. Additionally, a manual search was carried out based on the reference lists of included studies. Detailed information on the search strategy can be found in the appendix. Articles were included if they met the following inclusion criteria: (1) application of the EQ-5D-3L or 5L as a primary or secondary outcome measure; (2) the minimum age of the sample is specifically reported and it does not include participants younger than 65 years of age and (3) information on the feasibility of the EQ-5D-3L or 5L was reported at least as secondary analysis. Articles were excluded if they were not in English or German, not available in full text or of a wrong publication type, i.e. abstracts, proceedings, review articles and study protocols. No restrictions were imposed on the cognitive status of study participants. The literature search was updated in November 2020 applying the same criteria as outlined above.

### Screening and data extraction

Two reviewers (OM and LB) independently screened the title and abstract of all identified studies after electronic and manual removal of duplicates. At the end of each screening stage, discrepancies were discussed and, if necessary, a third reviewer (WG) was consulted to resolve any variance. Subsequently, full texts for appropriate articles were retrieved and assessed for eligibility based on pre-defined inclusion and exclusion criteria. A standardised data extraction form was used to guide a structured review process. The data extraction process was conducted by one reviewer (LB) and verified by the second reviewer (OM) to check for missing extractions. The following information was extracted for each study: author, year of publication, country, study type, EQ-5D version, mode of administration, number and age of participants. We further extracted characteristics, which are specific to older adults such as information on the functional status and the living arrangement.

During the search and extraction process of information related to the feasibility of the EQ-5D, we concentrated on information regarding the proportion and distribution of missing responses of both the descriptive system and EQ VAS to identify potentially problematic items. Derived from that, we examined the completion rate describing the proportion of computable index values, which is only viable if the EQ-5D health state information is complete on all five dimensions (excluding the EQ VAS) [11, 39]. We further screened for information on the time needed to complete all components of the EQ-5D, the administration mode as well as its appropriateness. Nonetheless, related aspects such as the ability to complete the measurement or problems during data collection process were also extracted.

## Results

### Study selection

**Fig. 1:**
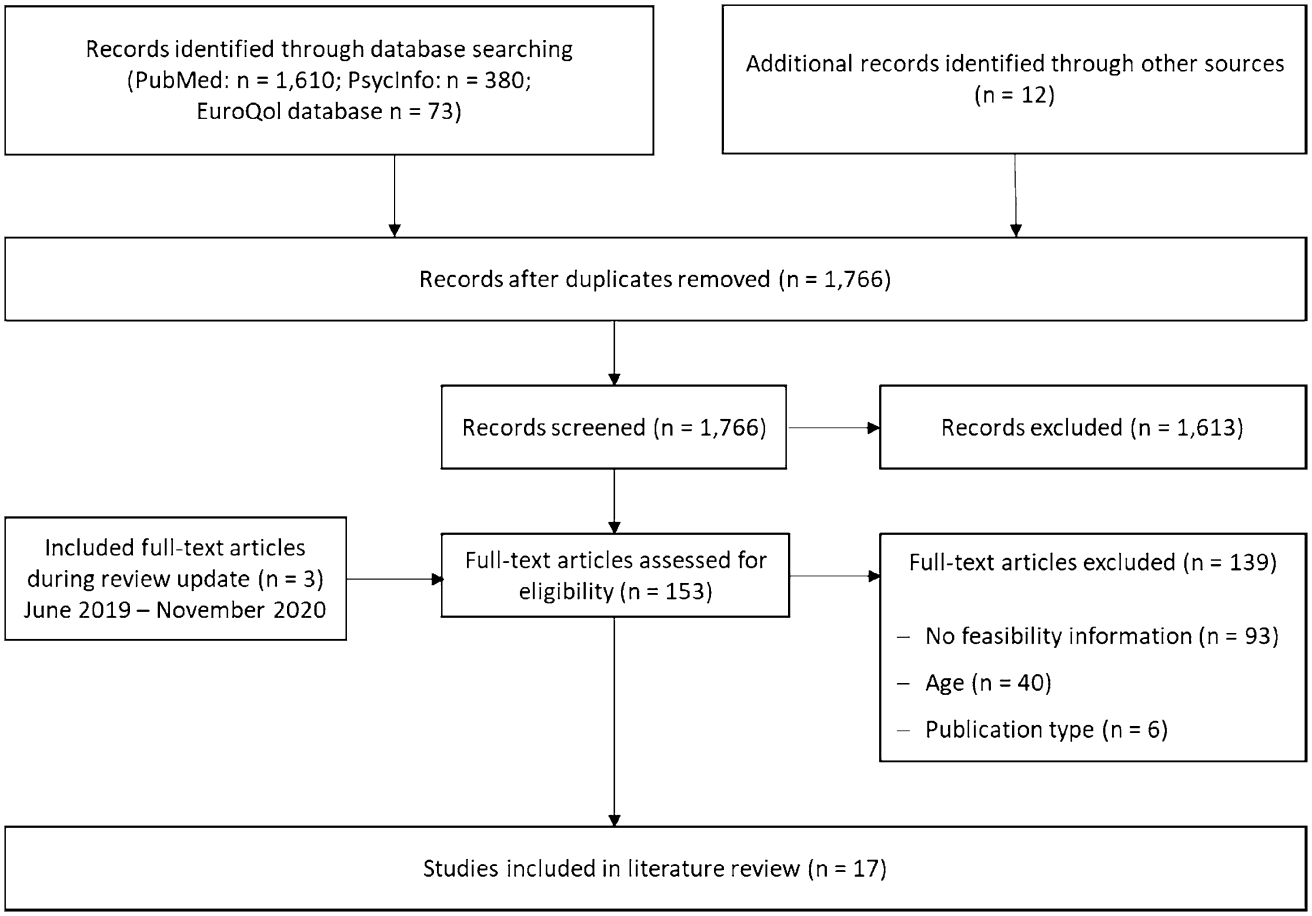
Flow chart of the literature search and study selection process

Our initial search including articles until June 2019 retrieved 2063 articles from the PubMed, PsycInfo and EuroQol website databases; 12 additional references were identified during the manual search of reference lists. After removal of duplicates, 1766 references were screened based on their titles and abstracts resulting in the exclusion of 1613 references. The remaining 153 studies were screened for eligibility in full text. Of those, 139 studies did not meet the inclusion criteria and, hence, were excluded. Main reasons for exclusion were the unavailability of feasibility information, insufficient age (sample not exclusively 65 years and over; only mean age reported) and publication type (no full text available; review article). The remaining 14 articles included in the review focus on aspects of the EQ-5D’s feasibility in the elderly population. The further conducted review update, including articles published between June 2019 and November 2020, resulted in three additional hits. Thus, the review eventually included 17 articles (Fig. 1).

### Study characteristics

Studies were mostly conducted in Europe except three studies from Australia, Canada and South Africa (Table 1). The reported sample size ranges from 10 to 3073 respondents with data being collected in both general (*n* = 4) and patient populations (*n* = 13). One study applied a qualitative approach to assess the feasibility of the EQ-5D-3L in older adults, whereas the remaining 16 studies used a quantitative study design (Table 2). Only two of the included studies used the EQ-5D-5L, whereas the 3L was used in the remaining 14 studies, with one study additionally using a cognitive bolt-on. The use of the EQ VAS is inconsistent; two studies make no statements towards its use, whilst one study explicitly states that the EQ VAS was not applied. The remaining 14 studies provide information based on the EQ VAS.

**Table 1:** Study characteristics of included studies

Reference, year	Study design	Country	Sample size—N	Study setting	Population	Living arrangement	Functional status	Percentage of women (N)	Mean age ± SD (range) in years
Arakawa Martins et al. [42]	Cross-sectional study	Australia	25	Care home	Patients ≥ 65 years with English speaking ability	Residential aged care sites	Inclusion: MMSE score > 22 IADL-scale (Lawton & Brody): 60% of participants were moderate to highly dependent for IADL 48% of participants had Charlson’s Comorbidities Index of ≥ 4	68% (17)	NR (66–100)
Botes et al. [45]	Cross-sectional study	South Africa Netherlands	60 (30 per country)	General population	Patients in South Africa and Netherlands ≥ 65 years	South Africa: home-dwelling Netherlands: one third of the sample each living independently, semi-independently in proximity to care and in residential aged care sites	NR	60%	75.2
Brazier et al. [20]	Randomised controlled trial	United Kingdom	377	General population	Women ≥ 75 years from the practice lists of four general practices	NR	NR	100%	80.1 ± 4.5 (NR)
Coast et al. [48]	Randomised controlled trial	United Kingdom	214	Rehabilitation	Patients ≥ 65 years admitted to a hospital trust with completed medical treatment and being suitable for rehabilitation	NR	Inclusion: MMSE score no cut-off 3% MMSE score ≤ 15 11% MMSE score 16–20 32% MMSE score 21–25 54% MMSE score 26–30 Barthel score assessed, but NR	70% (150)	79 (74–84)
Davis et al. [54]	Cross-sectional study	Canada	215	Falls Prevention Clinic	Patients ≥ 70 years who have visited a doctor after a non-syncopal fall within the last 12 month	Community-dwelling	Inclusion: MMSE score ≥ 24; mean MMSE score 26.9 (SD 3.4)	NR	79.3 ± 6.2
Grund et al. [40]	Cross-sectional study	Germany	86	Rehabilitation	Multimorbid geriatric in-patients from a geriatric rehabilitation clinic	NR	NR	69.8% (60)	80.98 ± 7.4
Hickson and Frost [47]	Cross-sectional study	United Kingdom	233	Hospital	Hospitalised elderly people ≥ 65 years receiving intense feeding support	NR	Inclusion: AMT score > 6 Barthel score assessed, but NR	55.4% (129)	NR
Holland et al. [43]	Randomised controlled trial	United Kingdom	145	Hospital	Patients ≥ 79 years who have been discharged from hospital and need to take two or more medications	Own home or warden controlled accommodation	Median AMT score 10	57%	84.7 (NR)
Hulme et al. [46]	Cross-sectional study	United Kingdom	73	Rehabilitation	Patients accepted to a rehabilitation program with complex physical, psychological and/or social difficulties, but medically stable	NR	Mean Barthel score 66.8 (SD 15.4)	78% (57)	82 (66–95)
Kunz [70]	Cluster-randomised trial	Germany	390	General medical	Patients ≥ 65 years who were supported by carers	Community-dwelling	Inclusion: MMSE score 10 – 24; mean MMSE score 18.6 (SD 3.8) Mean Barthel score 72.9 (SD 26.5)	67.5%	80.2 ± 6.7 (65–100)
Luthy et al. [41]	Cross-sectional study	Switzerland	3073	General population	Individuals ≥ 65 years N = 1.076 French speaking; N = 1.238 German speaking; N = 574 Italian speaking	Community-dwelling	NR	48.4% (1,399)	NR (65–90+)
Michalowsky et al. [50]	Cross-sectional study	Germany	NR	Dementia care network	Patients with dementia and their carers	Community-dwelling	Mean FAST stage 6.25 (SD 1.0); 94.3% of participants formally diagnosed with dementia; IADL-scale (Lawton & Brody): 31.5% of participants had no problems 43.6% of participants had moderate problems 24.9% of participants had severe problems	59.1% (243)	79 ± 8.5
Pérez-Ros and Martínez-Arnau [62]	Cross-sectional study	Spain	251	Care home	Participants > 70 years with cognitive impairment diagnosed by geriatrician	Residential aged care sites	Inclusion: MMSE score 10–24; mean MMSE score 15.6 (SD 5.2) Mean Barthel score 41.1 (SD 31.4)	76.9% (193)	84.6 ± 9.2 (70–104)
Pérez-Ros et al. [63]	Cross-sectional study	Spain	188	Primary care centre	Older adults > 70 years with cognitive impairment assessed by family physician	Community-dwelling	Inclusion: MMSE score 10–24; mean MMSE score 21.2 (SD 3.2) Mean Barthel score 88.5 (SD 17.3)	64.9% (122)	79.2 ± 5.2 (70–95)
Tidermark et al. [71]	Prospective cohort study	Sweden	90	Hospital	Patients ≥ 65 years with a femoral neck fracture after a fall and unhindered walking ability	Living independently	Inclusion: SPMSQ score ≥ 3; Mean SPMSQ score 8.2; median 9; range 3–10 ADL-scale (Katz): A–B 94% of participants C–G 3% of participants	51%	80 ± 7.3 (66–92)
van Laar et al. [55]	Longitudinal study	Netherlands	138 (83.3%)	Hospital	Patients ≥ 75 undergoing cardiac surgery	NR	NR	61%	79.5 ± 2.8 (75.1–87.5)
van Leeuwen et al. [44]	Qualitative study	Netherlands	10	General population	Elderly general population ≥ 65 years with at least two health issues	Community-dwelling	Exclusion: cognitive impairment or impaired mental status (assessment scale NR) Mean PRISMA-7 score 4; median 4; range 1- 6	60%	NR (67–100)

*NR* not reported, *N* sample size, *SD* standard deviation, *MMSE* mini-mental state examination, *AMT* abbreviated mental test, *FAST* functional assessment staging of Alzheimer’s disease, *IADL* instrumental activities of daily living, *SPSMQ* short portable mental status questionnaire, *ADL* activities of daily living, *PRISMA-7* program of research on integration of services for the maintenance of autonomy

**Table 2:** EQ-5D characteristics and extracted feasibility information

Reference, year	EQ-5D version	Mode of questionnaire administration	Missing values	Completion rate EQindex	Statements towards completion	Completion time
Arakawa Martins et al. [42]	EQ-5D-5L (use of EQ VAS unclear)	Self-complete version on paper; under observation			Comprehension issue: N = 1 (out of 25)—4%	Total time (mean ± SD) 265 ± 158 s
Botes et al. [45]	EQ-5D-3L + C	Self-complete version on paper; interviewer support if needed	No missing values		“…the elderly performed the health state description […] with ease”	
Brazier et al. [20]	EQ-5D-3L	Self-complete version on paper	Below 10% on the descriptive system and EQ VAS		“Brief and easy to use in this age group”	
Coast et al. [48]	EQ-5D-3L	Self-complete version on paper; interviewer support if needed	At baseline: MO—4.2% SC—2.3% UA—3.3% PD—2.3% AD—3.3% EQ VAS—16.4% 4-week follow-up MO—9.5% SC—7.5% UA—7.0% PD—7.5% AD—7.5% EQ VAS—20.1%	At baseline 93.9% 4-week follow-up 88.9%	Around 50% required interviewer administration; Probability of requiring interviewer administration at 65 years is 11%; at 75 years is 37% and at age 85 is 73%	
Davis et al. [54]	EQ-5D-3L	Self-complete version on paper	MO—0% SC—0% UA—0.5% PD—0% AD—0.5% EQ VAS—2.3%	99.1%		
Grund et al. [40]	EQ-5D-5L	Self-complete version on paper; interviewer support if needed	UA—10.5%	89.5%	All patients able to answer EQ VAS Usual activities caused most comprehension problems (N = 15) “EQ-5D-5L can be handled quickly and without major complications” Elderly people seem to omit items which may not be relevant to them	Total sample (mean ± SD) 222 ± 117 s No assistance group (mean ± SD) 182 ± 105 s Some assistance group (mean ± SD) 255 ± 138 s Strong assistance group (mean ± SD) 186 ± 70 s
Hickson and Frost [47]	EQ-5D-3L	Self-complete version; interviewer support if needed	EQ VAS—25.3%	82.8%	Concept of EQ VAS was difficult to understand wording of the descriptive system found to be restrictive	
Holland et al. [43]	EQ-5D-3L	self-complete version on paper; interviewer support if needed		Baseline—95.9% 81% over three time points	Anxiety/depression caused some embarrassment EQ VAS caused most difficulty	Less than 5 min
Hulme et al. [46]	EQ-5D-3L	Interviewer administered; four respondents self-completed the EQ-5D	5.5–6.8% in each dimension EQ VAS—8.2%	93.1%	Difficulty with completing and understanding the EQ VAS (27%) wording of the descriptive system: - limited understanding - found to be restrictive 55% asked for help, explanation or clarification on one or more of the five items Difficulty understanding the concept of today’s health	
Kunz [70]	EQ-5D-3L (EQ VAS not included)	Not specifically reported	MO—4.6% SC—4.6% UA—5.4% PD—4.6% AD—4.6%	94.6%		
Luthy et al. [41]	EQ-5D-3L	Computer-assisted personal interview		94% (total sample) 65–69–94.9% 70–74–95.3% 75–79–93.7% 80–84–95% 85–89–92.7% 90 and above–90.9%		
Michalowsky et al. [50]	EQ-5D-3L	Self-complete version; interviewer support if needed	MO—8.8% SC—10% UA—10.7% PD—9.2% AD—9.8% EQ VAS—18%	88.9%		
Pérez-Ros and Martínez-Arnau [62]	EQ-5D-3L	Self-complete version; interviewer support if needed	No missing values in the analysis sample	100% in the analysis sample		
			N = 14 (5.3%) were not capable to respond to the survey items—EQ-5D-3L causality unclear			
Pérez-Ros et al. [63]	EQ-5D-3L	Self-complete version; interviewer support if needed	No missing values in the analysis sample	100% in the analysis sample		
			N = 20 (11.5%) were not capable to respond to the survey items—EQ-5D-3L causality unclear			
Tidermark et al. [71]	EQ-5D-3L	Not specifically reported		Inclusion—100% 4-months—97% 17-months—89%		
van Laar et al. [55]	EQ-5D-3L	Self-complete version on paper; postal survey at follow-up		Baseline—83.3% 1 year post-operative—69% 2 year post-operative—58%		
van Leeuwen et al. [44]	EQ-5D-3L (use of EQ VAS unclear)	Self-complete version; interviewer support if needed			Narrow item interpretation UA, PD & AD Positive answering in all dimensions except for SC Low degree of problems with mapping of response to the descriptive system Comprehensibility: Most easy to answer; specific questions; clear response options	

*SD* standard deviation, *N* sample size, *S* seconds, *MO* mobility, *SC* self-care, *UA* usual activities, *PD* pain or discomfort, *AD* anxiety or depression, *EQ VAS* EQ-5D visual analogue scale

With regard to the administration mode two studies did not make any specific statements as to how the EQ-5D was administered (Table 2). The majority of studies (*n* = 13) applied a self-complete version of the EQ-5D to collect HRQoL data, where interviewer support was available on request in nine of those studies. Moreover, two of the included studies applied an interviewer-based approach to collect EQ-5D data in the elderly population.

### Feasibility information

We included 17 studies examining aspects of the EQ-5D’s feasibility in the elderly. However, only eight studies referred to this topic as “feasibility”, whilst another seven studies referred to this under the term “completion” and one further study each investigated these properties labelled as “practicality” or “acceptability”. Included studies assessed the EQ-5D’s feasibility in terms of missing values (*n* = 11), completion rates (*n* = 13), made qualitative statements towards the completion (*n* = 9) and measured the time required to complete the EQ-5D (*n* = 3). One study investigated all four aspects, whilst four studies reported results on three of these aspects; six studies examined at least two feasibility outcomes, whilst six studies only reported on one of the feasibility aspects (Table 2).

Detailed information on missing data for the EQ-5D-3L was reported by five studies, whilst three additional studies only report that missing values did not exceed 10% in general. At the dimension-level, the proportion of missing values did not exceed 10.7% across all five dimensions. In addition, information on missing values for the 5L descriptive system was only provided by Grund et al. [40], where only responses to the usual activities dimension were missing in 10.5% of the cases. Missing values on the EQ VAS were evaluated in nine studies. Three studies found no missing values and one study reported that less than 10% were missing. Further, five studies reported the specific proportion of missing responses to the EQ VAS ranging from 2.3 up to 25.3%, generally exceeding the share of missing values on the descriptive system.

Completion of the EQ-5D-3L was either around but mostly above 90% for the baseline assessment, except in two studies which reported a completion of just above 80%. Luthy et al. [41] provided age-specific completion rates of 94% for the total sample, still achieving above 90% for respondents aged 90 years and above. Again, only Grund et al. [40] reported a completion rate of 89.5% for the EQ-5D-5L.

Completion time was assessed in three studies. Both the EQ-5D-3L and EQ-5D-5L can usually be completed in less than five minutes (more details in Table 2) [40, 42, 43]. As can be assumed, respondents self-completing the EQ-5D need less time to complete than respondents who are in need of assistance, but were found to need similar amounts of time than respondents with a strong need for assistance, essentially administering the EQ-5D-5L in an interviewer-based approach [40].

Moreover, nine studies described the completion of the EQ-5D qualitatively. Overall, the measures were found to be brief and easy to use [20, 40, 44, 45]. Comprehension issues were rarely reported, but related to narrow item interpretation or restrictive item wording, where only few respondents had problems mapping their response to the descriptive system [42, 44, 46, 47]. Moreover, comprehension issues with regard to the EQ VAS were also reported in three studies [43, 46, 47], where Hulme et al. [46] stated that 27% of respondents had trouble completing or understanding the EQ VAS.

Coast et al. [48] assessed whether respondents required an interviewer-based approach controlling for age. In total, 50% of their sample required an interviewer to complete the EQ-5D-3L; whilst stratifying for age the probability of requiring interviewer administration was at 11% at 65 years, 37% at 75 years and at 73% at age 85. At a similar level Hulme et al. [46] found that 55% required additional help from an interviewer whilst answering the EQ-5D-3L.

## Discussion

Over the past three decades the EQ-5D has been applied in an extensive list of populations and settings and amongst those elderly populations and patients were frequently examined, too. Even though the EQ-5D’s feasibility properties were confirmed and found to be unproblematic for the overall general population by two recent major reviews [22, 23], feasibility of the EQ-5D is not systematically explored for the elderly population and warrants further examination [18, 49]. Therefore, this review summarised the available information on the feasibility properties of both the EQ-5D-3L and EQ-5D-5L in the elderly population. In the light of the reviewed studies, the terminology around this measurement property and which aspects of feasibility are commonly reported are inconsistently used. Overall, missing values of approximately up to 10% on the descriptive system and completion rates of around 90% seem to be ballpark figures in elderly populations. On the other hand, completion of the EQ VAS seems to pose a higher burden to older respondents, since missing rates are generally higher. Also of interest is the high prevalence of interviewer-assisted or even interviewer-based administration of the EQ-5D in the older population.

This review identified several different synonyms for feasibility such as ‘completion’, ‘practicality’ or ‘acceptability’. However, all labels aim to describe the applicability of the measure to the target population in a similar manner, viz. how conveniently and successfully the measure can be completed. Yet, to the best of our knowledge, none of the leading QoL organisations such as the EuroQol group, the international society for quality of life research (ISOQOL), World Health Organization (WHO) or the European Organisation for Research and Treatment of Cancer (EORTC) Quality of Life Group provide a definition of feasibility. To aid the standardisation of reporting this measurement property and the usability of available evidence for researchers, we suggest summarising these labels under the term ‘feasibility’.

Regardless of the label used in individual studies, there was little agreement on how feasibility was operationalised and therefore included studies examined feasibility in terms of missing values, completion rate, time required to complete the EQ-5D or—more broadly—in qualitative statements referring to the completion of the instrument. However, the amount of available information on each of these parameters varied greatly. One of the more frequently reported outcomes were missing values. Overall, the proportion of missing values was mostly below 7% on the five dimensions with few exceptions, e.g. in a study by Michalowsky et al. [50] including cognitively impaired respondents; but generally missing values did not exceed 11%. Where detailed information on the dimension-level was available, those suggested that dimensions were equally affected with no particular one sticking out. Two recent systematic reviews provide a reference value of 5% missing values for both the EQ-5D-3L and EQ-5D-5L [22, 23]. This upper value largely seems to hold for the included studies with only a marginal excess effect in missing values in the elderly population, which was already described elsewhere [30, 51–53]. Even though the proportion of missing values seems to increase slightly with higher age, the EQ-5D compared more favourably in terms of missing values when compared to other instruments that are commonly applied with the elderly such as the SF-36 [20, 40], the AQoL [43] or ICECAP-O [54].

A related concept is the completion rate of the descriptive system. Findings from this review suggest that the proportion of incomplete responses is commonly less than 10%, i.e. more than 90% of index values are computable. Considerably lower completion rates were reported by Hickson and Frost [47], arguing that patients were too ill to complete the questionnaire. Also, van Laar et al. [55] report an equally low completion rate of 83.3%, however, it remains unclear to what extent the study design or the disease negatively interfered with completion rates. Again, comparing our findings with the benchmark completion rates of more than 93.4% (3L) and 96% (5L) provided by Buchholz et al. [22], we find that the share of computable index values seems to slightly decrease in the elderly population. This is congruent with findings from Luthy et al. [41] who found that completion rates for the EQ-5D-3L were negatively associated with increasing age. However, this finding is not surprising given that missing values were also slightly more prevalent in the elderly, which in turn lead to incomplete health state information. Based on the reviewed evidence on missing values and completion rates of the descriptive system, we would like to argue that a magnitude of approximately less than 10% missing values and about 90% of computable EQ-5D index values demonstrate reasonable feasibility of the EQ-5D in the elderly population.

A further component that was found to be more frequently missing was the EQ VAS. The range of missing EQ VAS ratings was 2.3–25.3% and differs considerably, but generally exceeds the proportion of missing values in the descriptive system. There is evidence that the concept of the EQ VAS is more difficult to comprehend than the descriptive system and causes the most problems [43, 46–48], which was found in older populations before [26]. Then again, higher proportions of missing EQ VAS ratings [32] and execution problems [56] were also reported for the younger populations, yet to a lesser extent.

Generally, missing data on both components the EQ VAS and the descriptive system diminish the available sample size for analyses and, if item nonresponse occurs systematically, this may result in biased results [57]. The decision on how to handle missing data in the analysis should be guided and justified based on the mechanism of missing data rather than the proportion of missing values per se [21, 39, 58–60]. Generally, preventing missing data before they occur is more efficient than an analytical remedy [61]. In this sense, a re-occurring topic in included studies is the high proportion of older respondents requiring assistance to complete the EQ-5D or even an interviewer-based approach [40, 46, 48]. Several issues, such as asking for explanations and clarifications due to limited item interpretation [40, 46, 48], were identified that may warrant interviewer support when collecting EQ-5D data in the elderly [44]. Similar findings were presented from a qualitative study with younger hip fracture patients (60+ years) by Rohr et al. [37] arguing that without interviewer support missing values would be significantly higher. At least three of the included studies acknowledged that interviewer support was needed, but did not further assess the amount of support that was required by the participants [47, 62, 63]. With regard to this, Coast et al. [48] report age-dependent probabilities of requiring interviewer assistance, further suggesting an increased need of assistance with higher age. On the other hand, several studies report good feasibility properties in the older population relying on self-completed measures without assistance [20, 42, 64], whereby the EQ-5D appears to be well applicable as a self-report measure in the elderly. Even though interviewer support was frequently provided in included studies, the effect of interviewer assistance on preventing missing values on the descriptive system and EQ VAS or increased completion rates cannot be quantified based on the available evidence for two reasons. First, included studies did not compare feasibility aspects between assisted and non-assisted respondent sub-groups and, secondly, studies were too heterogeneous in their outcomes and study characteristics to identify factors that facilitate adequate feasibility. Furthermore, adopting an interview approach is resource intensive and may introduce additional bias or measurement error, if implemented improperly and, thus, may offset potential benefits [65]. The controversial evidence and the lack of studies exploring the effect of an interviewer approach express the need for further research on this topic. Future qualitative research as done by Rohr et al. [37] and van Leeuwen et al. [44] may help to better understand the role of interviewers in the data collection process. Alternatively, a cognitive de-briefing of interviewers in quantitative studies may shed a light on the heterogeneity of the elderly and help exploring sub-groups that might benefit from interviewer assistance.

From our review, we found only two of the included studies applied the EQ-5D-5L in the elderly population and analysed aspects of the instrument’s feasibility, which limits the generalisability of our findings beyond these two studies for the EQ-5D-5L. However, we would expect the EQ-5D-5L’s feasibility to be comparable or potentially even better than those of the EQ-5D-3L, since the EQ-5D-5L kept the brevity. Further, enabling respondents to better map their health onto the descriptive system in more detailed distinctions might potentially increase engagement to the task [66]. But then again, this may have also increased the cognitive burden hampering instrument completion and data quality [67–69], however, not to an extent which is believed to overburden respondents [66]. Since none of the included studies comparatively examined both the EQ-5D-3L and EQ-5D-5L in the elderly population, this gap warrants further research.

Some limitations of this study should be considered. In accordance with the underlying age definition for the elderly population, this study only included publications, which focussed on study populations exclusively being 65 years and above. Due to this strict age-related eligibility criterion, some studies were excluded where the majority of respondents may have met the inclusion criteria, whilst only a minor share of respondents was not eligible. Also, included studies exclusively sampled respondents from western and developed countries, which does not allow any generalisation on the feasibility in elderly populations in Asia, Africa or South America, where the older population may be assumed to be different due to their cultural believes or lower literacy in rural regions. Furthermore, the heterogeneity of the target population, which may include healthy and independently living respondents, but also frail or cognitively impaired participants, also limits the generalisability of our findings, especially with regard to the EQ-5D-5L, which was only included in two studies. Moreover, despite searching several databases, we might have missed relevant publications due to an inconsistent use of terminology in addressing feasibility and more importantly due to different search engines for the databases, which may have partially prevented the identification of studies addressing feasibility as a secondary or even only as a descriptive analysis. As a potential remedy to this limitation, we searched all reference lists of included articles to mitigate this risk.

## Conclusion

This study aimed to assess the EQ-5D’s feasibility properties when used in the elderly population. Our findings suggest that missing values or comprehension problems—especially with the EQ VAS—are slightly more prevalent in older age groups compared to the younger general population. However, these aspects are well within an acceptable range and still considerably lower than in other measures such as SF-36 or ICECAP-O, which are frequently used in the elderly. Furthermore, older respondents seem to have a higher propensity of requiring some degree of assistance or even an interviewer-based approach. Overall, evidence from the reviewed literature indicates that the EQ-5D-3L has good feasibility properties and, hence, is highly applicable in older respondents. Moreover, further research is needed to explore feasibility properties of the EQ-5D-5L in older respondents too, whilst examining the proportion of missing values, completion rate and completion time considering the role of any interviewer support in the data collection process.
